# Overexpression of Brg1 Alleviates Hepatic Ischemia/Reperfusion-Induced Acute Lung Injury through Antioxidative Stress Effects

**DOI:** 10.1155/2017/8787392

**Published:** 2017-07-16

**Authors:** Mian Ge, Chaojin Chen, Weifeng Yao, Shaoli Zhou, Fei Huang, Jun Cai, Ziqing Hei

**Affiliations:** Department of Anesthesiology, The Third Affiliated Hospital of Sun Yat-sen University, Guangzhou, Guangdong 510630, China

## Abstract

**Aim:**

To investigate whether overexpression of Brahma-related gene-1 (Brg1) can alleviate lung injury induced by hepatic ischemia/reperfusion (HIR) and its precise mechanism.

**Methods:**

Cytomegalovirus-transgenic Brg1-overexpressing (CMV-Brg1) mice and wild-type (WT) C57BL/6 mice underwent HIR. Lung histology, oxidative injury markers, and antioxidant enzyme concentrations in the lung were assessed. The protein expression levels of Brg1, nuclear factor erythroid 2-related factor 2 (Nrf2), heme oxygenase-1 (HO-1), and NAD(P)H:quinone oxidoreductase 1 (NQO1) in the lung were analyzed by Western blotting.

**Results:**

In the WT group, histopathological analysis revealed that lung damage peaked at 6 h after HIR. Meanwhile, the lung reactive oxygen species (ROS) and 8-isoprostane levels were significantly increased. The protein expression of Brg1 in lung tissue decreased to a minimum at 6 h. Overexpression of Brg1 alleviated lung injury and decreased the amounts of oxidative products, including the levels of 8-isoprostane and ROS, as well as the percentage of positive cells for 4-hydroxynonenal (4-HNE) and 8-oxo-2′-deoxyguanosine (8-OHdG). Brg1 overexpression increased the expression and nuclear translocation of Nrf2 as well as activated the antioxidases. In addition, it decreased the expression of inflammatory factors.

**Conclusion:**

Overexpression of Brg1 alleviates oxidative lung injury induced by HIR, likely through the Nrf2 pathway.

## 1. Introduction

Hepatic ischemia/reperfusion injury (HIRI) occurs in many surgeries, including liver transplantation, liver resection, and cardiovascular surgery [1, 2]. HIRI not only leads to liver injury but also causes remote organ damage, especially lung injury [3, 4]. We have reported previously that up to 58.2% of patients developed pulmonary complications after liver transplantation; among them, 27.5% suffered from an acute lung injury (ALI) [5, 6]. Given that numerous risk factors are involved in ALI induced by hepatic ischemia/reperfusion (HIR), effective and preventive strategies are needed [7, 8].

Multiple factors are reported to take part in the pathogenesis of post-HIR ALI [9]. Excessive reactive oxygen species (ROS) produced in the liver after ischemia/reperfusion is one of the main mechanisms involved in HIR-induced ALI [10–12]. Excessive production of ROS causes ALI by oxidative stress [13, 14], apoptosis [15–17], and inflammatory responses [18]. Thus, clearance of ROS appears to be a potential therapeutic strategy for post-HIR ALI.

Physiologically, every cell is able to defend itself against the oxidative damage caused by excessive ROS triggering the delivery of antioxidant enzymes [19]. Cellular antioxidant enzymes, including catalase, glutathione S-transferase (GST), thioredoxin 2, superoxide dismutase (SOD), glutathione peroxidase (GPx), heme oxygenase-1 (HO-1), and NAD(P)H:quinone oxidoreductase 1 (NQO1), constitute the first line of defense against the toxic effects of ROS [20, 21]. Nuclear factor erythroid 2-related factor 2 (Nrf2) is an important transcription factor that is known to act as a master regulator of the antioxidant response [21]. Following exposure of cells to oxidative stress, Nrf2 is induced and translocated into the nucleus to activate the expression of a series of antioxidative and cytoprotective proteins that include GPx, glutamate-cysteine ligase (GCL), NQO1, and HO-1 [22, 23]. However, we have found previously that an increased expression of Nrf2 in liver tissue after liver transplantation did not effectively activate the expression and activity of antioxidant enzymes at an early reperfusion time. This finding indicated that the Nrf2-induced antioxidant response was restricted by post liver transplantation mechanisms. However, those mechanisms inhibiting Nrf2 induction after reperfusion of the liver and their importance in combating ALI remain unclear.

Being a catalytic subunit of SWI2/SNF2-like chromatin-remodeling complexes, Brahma-related gene-1 (Brg1) disrupts the chromatin architecture of target promoters and has been reported not only to play an important role in gene transcription and expression but also to regulate stem cell proliferation and differentiation in different tissues and cells in humans and animals, as well as the process of disease development [24]. Zhang et al. have reported that Brg1 can interact with Nrf2 and is specifically required for Nrf2-mediated activation of HO-1 gene transcription in multiple tumor cells [25]. However, whether Brg1 interacts with Nrf2 and mediates the activation of other antioxidant enzymes in the development of HIR-induced ALI is still unclear. Based on the above-mentioned findings, we hypothesized that upregulation of Brg1 expression could promote the activation of Nrf2-mediated antioxidant enzymes and alleviate lung injury induced by HIR.

## 2. Methods

### 2.1. Animals and Treatment

The experimental protocols and design were approved by the Institutional Animal Care and Use Committee at Sun Yat-sen University. Eight- to ten-week-old C57BL/6 male mice were used. Animal care was conducted in accordance with the Guidelines of Sun Yat-sen University for Animal Experimentation. Cytomegalovirus-transgenic Brg1-overexpressing (CMV-Brg1) mice (Cyagen Biosciences, ID: TGBS130618AG1, USA) with the C57BL/6 background were used to generate CMV-Brg1 and wild-type (WT) littermates. As described previously [26], genotyping was performed by polymerase chain reaction using genomic DNA extracted from a tail snip. The mice were randomly assigned into five groups (*n* = 8 per group) in the initial intervention model establishment study, which included sham-operated as well as 3, 6, 12, and 24 h reperfusion groups. Subsequent in vivo studies were performed using the 6 h reperfusion HIR model. Both the CMV-Brg1 and WT mice were then randomly assigned into two groups (*n* = 5 per group): sham-operated group (sham) and HIR group.

### 2.2. HIR Model

In order to minimize investigator bias, the operator was blinded to the treatment conditions. All the mice were anesthetized by an intraperitoneal injection of ketamine (60 mg/kg) and then received left and median liver lobe clamping for 60 min and reperfusion for the designated time. The sham group was treated in an identical manner to the surgery group except it was not subjected to renal pedicle occlusion. After the operation, the mice were housed in microisolator cages and allowed free access to water and chow. After a designated reperfusion time, mouse blood and lungs were collected for further experiments.

### 2.3. Hematoxylin-Eosin Staining and Histopathological Analysis

At the end of the experiment, the mice were anesthetized by an intraperitoneal injection of 60 mg/kg ketamine. Following exposure, the left lung was removed and perfused with PBS and then fixed in 10% buffered formalin overnight at 4°C. The lung tissues were then embedded with paraffin, sectioned at 5 *μ*m thickness, and stained with hematoxylin and eosin. Histological severity of lung injury was graded using a 0 to 4 point scale according to combined assessments of edema, neutrophil infiltration, hemorrhage, bronchiole epithelial desquamation, and hyaline membrane formation as follows: 0, no injury; 1, modest injury (including limited interstitial edema and congestion, but no interstitial neutrophilic infiltration with inflammatory cells in the alveolar spaces); 2, intermediate injury (including mild interstitial edema, congestion, and interstitial neutrophilic infiltration with inflammatory cells in the alveolar spaces); 3, widespread injury (including more prominent congestion and interstitial edema with neutrophils partially filling the alveolar spaces); and 4, widespread injury (including most prominent, marked congestion and interstitial edema with neutrophilic infiltration nearly filling the alveolar spaces or with pulmonary consolidation). A total of six fields were randomly selected for each slide, and the average was used as the histopathological score [27].

### 2.4. ROS Detection

As reported previously [28, 29], the accumulation of ROS in lung tissue was estimated by using the in vitro reactive nitrogen species assay kit OxiSelect (Cell Biolabs, San Diego, CA, USA), according to the manufacturer's protocol.

### 2.5. Detection of 8-Isoprostane, Glutamate-Cysteine Ligase Catalytic Subunit (GCLC), Superoxide Dismutase (SOD), Glutathione S-Transferase Alpha 1 (GST*α*1), Interleukin-6 (IL-6), and Tumor Necrosis Factor-*α* (TNF-*α*)

Lung tissue was transformed into 10% homogenates with frozen normal saline and spun at 3000 rpm for 10 min. The pulmonary protein content was measured using a Bicinchoninic Acid Protein Assay Kit (KeyGen Biotech Company, Nanjing, China). The concentrations of 8-isoprostane (Cayman Chemical Company, USA), GCLC (USCN Business Co. Ltd., Wuhan, China), GST*α*1 (USCN Business Co. Ltd., Wuhan, China.), SOD (KeyGen Biotech Company, Nanjing, China.), IL-6 (USCN Business Co. Ltd., Wuhan, China), and TNF-*α* (USCN Business Co. Ltd., Wuhan, China) were measured by enzyme-linked immunosorbent assay kits. All detections were performed according to the assay kit manufacturer's instructions.

### 2.6. Immunofluorescence and Immunohistochemistry

5 *μ*m thick HIR-injured lung sections embedded in paraffin and cells grown on sterile coverslips and then subjected to the HIR protocol were prepared for immunofluorescence assays. The samples were stained with anti-8-OHdG antibody (1 : 200; LS-C415095, LSBio) at 4°C overnight, followed by anti-rabbit or anti-mouse IgG (1 : 1000), according to the manufacturer's instructions. Cell nuclei were stained with DAPI. All images were captured using an EVOS FL fluorescence microscope (EVOS FL, Life Technology), and 10 randomly selected fields of each sample were semiquantified.

Immunohistochemical staining was performed to detect 4-hydroxynonenal (4-HNE) and Nrf2 expression in lung sections embedded in paraffin with anti-4-HNE antibody (1 : 100; ab46545, Abcam) and anti-Nrf2 antibody (1 : 100, AP52269, Abgent), respectively.

### 2.7. Western Blot Analysis

Western blotting was performed following standard procedures. Anti-Nrf2 antibody (1 : 100, AP52269, Abgent), anti-Brg1 antibody (1 : 100; ab110641, Abcam), anti-NQO1 antibody (1 : 100; ab28947, Abcam), anti-HO-1 antibody (1 : 100; sc-10,789, Santa Cruz), and secondary antibody (1 : 2000; Millipore) were used to detect protein expression. Anti-*β*-actin and anti-lamin B2 (Proteintech) were used at 1 : 5000. Images were acquired by a Tanon 5500 imaging system (Tanon, Shanghai). The images were scanned with the ImageJ scanning software, and the data were expressed as relative values to sham or control values.

### 2.8. Statistical Analysis

Statistical analysis was performed using SPSS 13.0 (SPSS Inc., Chicago, IL, USA) and Sigmaplot 10.0 (Systat Software Inc., Chicago, IL, USA). Data normality was tested using the Kolmogorov-Smirnov test. Multiple comparisons among different groups were analyzed using one-way analysis of variance, followed by Tukey's post hoc test. Quantitative data are presented as mean ± standard error of the mean. *P* values less than 0.05 were considered statistically significant.

## 3. Results

### 3.1. HIR Induces Oxidative Stress and ALI in the WT Mice

**Figure 1:**
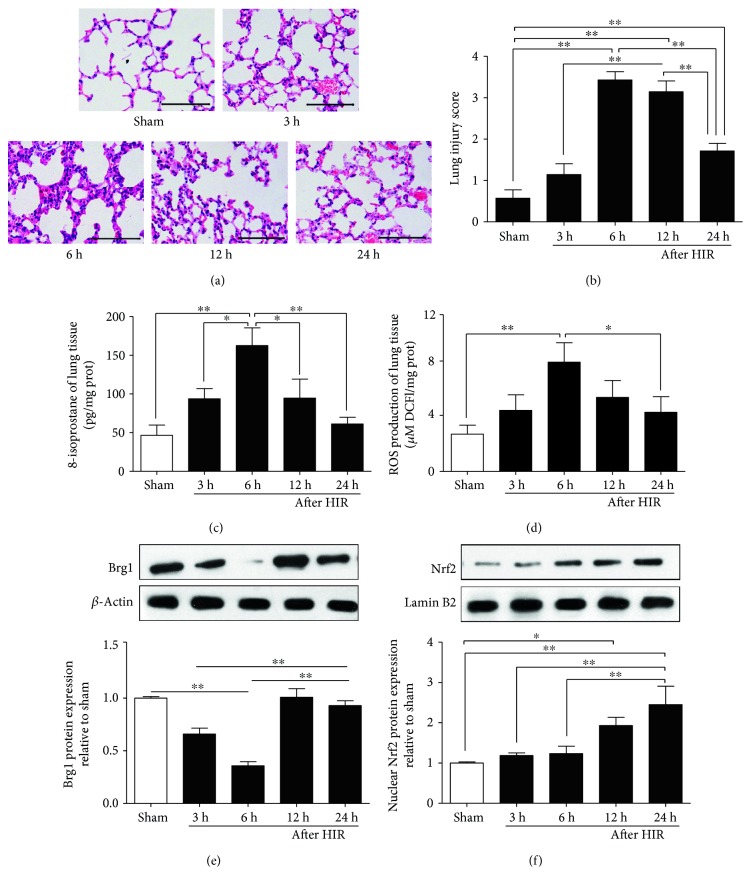
Hepatic ischemia/reperfusion (HIR) induces oxidative stress and acute lung injury at different time points (3, 6, 12, and 24 h) after HIR in the wild-type mice. (a) Hematoxylin-eosin staining of lung sections (×200); (b) lung pathological scores; (c) and (d) levels of 8-isoprostane and reactive oxygen species (ROS); (e) Brg1 protein expression in lung tissue; and (f) Nrf2 protein expression in lung tissue; ^∗^*P* < 0.05; ^∗∗^*P* < 0.01.

After the process of HIR, pathological damage was detected in the lungs (Figure 1(a)), including increased alveolar damage, perivascular and peribronchial edema, alveolar wall capillary hyperemia, and pulmonary interstitial inflammatory cell infiltration. It reached a peak at 6 h after reperfusion (Figure 1(a)), as reflected by the significant increase in pathological scores (Figure 1(b)), and then was restored gradually to normal.

As shown in Figures 1(c) and 1(d), the changes of ROS and 8-isoprostane levels in lung tissues were consistent with lung pathological damage. Similarly, the Brg1 protein expression in lung tissue began to decline after reperfusion, significantly reduced at 3 h and 6 h (*P* < 0.01 versus the Sham group), reached a minimum at 6 h, and then gradually increased at 12 h and 24 h (Figure 1(e)). Nrf2 protein expression in the lung tissue did not significantly change within the first 6 h after reperfusion, but it increased significantly at 12 h and 24 h after reperfusion (Figure 1(f)).

### 3.2. Brg1 Overexpression Protects against ALI and Oxidative Stress Induced by HIR

HIR resulted in severe lung pathological injury that was associated with significant oxidative damage and a significant decrease in Brg1 expression, suggesting that there might be an important role for Brg1 in ALI induced by HIR. To further explore this role, the CMV-Brg1 and WT mice were used to develop an appropriate HIR model.

**Figure 2:**
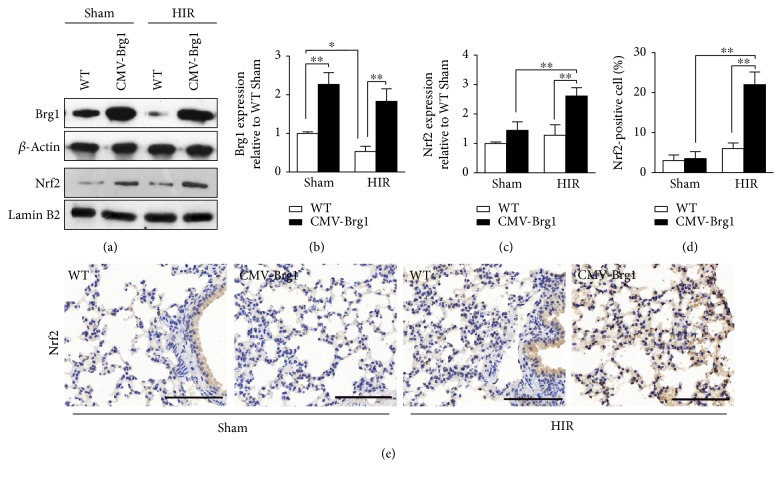
Association of Brg1 overexpression with increased nuclear transfer of Nrf2. (a–c) The protein expression levels of Brg1 and Nrf2 were detected by Western blot in the CMV-Brg1 and WT mice after HIR. (d, e) Immunohistochemical staining (×200) of lung Nrf2 and densitometric analysis showed that the Nrf2-positive cells in lung tissue of the CMV-Brg1 mice were more pronounced. ^∗^*P* < 0.05; ^∗∗^*P* < 0.01.

**Figure 3:**
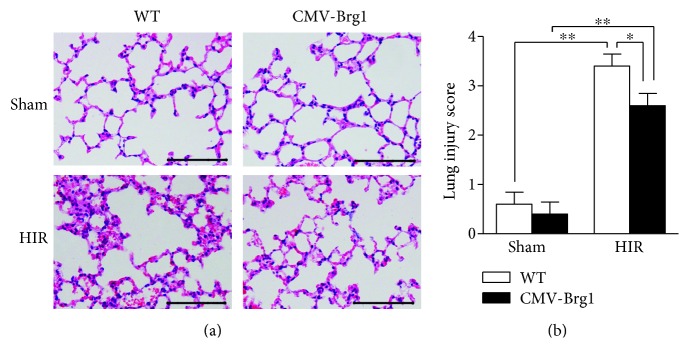
Overexpression of Brg1 protects against acute lung injury and oxidative stress induced by HIR. Cytomegalovirus-transgenic Brahma-related gene-1-overexpressing (CMV-Brg1) mice and wild-type (WT) mice were subjected to sham operation or HIR. (a) Hematoxylin-eosin staining of lung sections (×200); (b) pathological scores of lung tissue. ^∗^*P* < 0.05; ^∗∗^*P* < 0.01.

As shown in Figure 2(a), the Brg1 expression level of the CMV-Brg1 mice was increased by 2.2-fold, compared with the WT mice. The pathological results showed that increased alveolar damage, perivascular and peribronchial edema, alveolar wall capillary hyperemia, and pulmonary interstitial inflammatory cell infiltration were present after HIR in the WT mice (Figure 3(a)). In contrast, after HIR, the CMV-Brg1 mice suffered only mild alveolar damage, mild hyperemia in the alveolar wall capillaries, and less infiltration of inflammatory cells than the WT mice (Figure 3(a)). Compared with the WT mice, the pathological damage, as further assessed by the pathological scores, was therefore significantly reduced in the CMV-Brg1 mice (Figures 3(a) and 3(b)).

**Figure 4:**
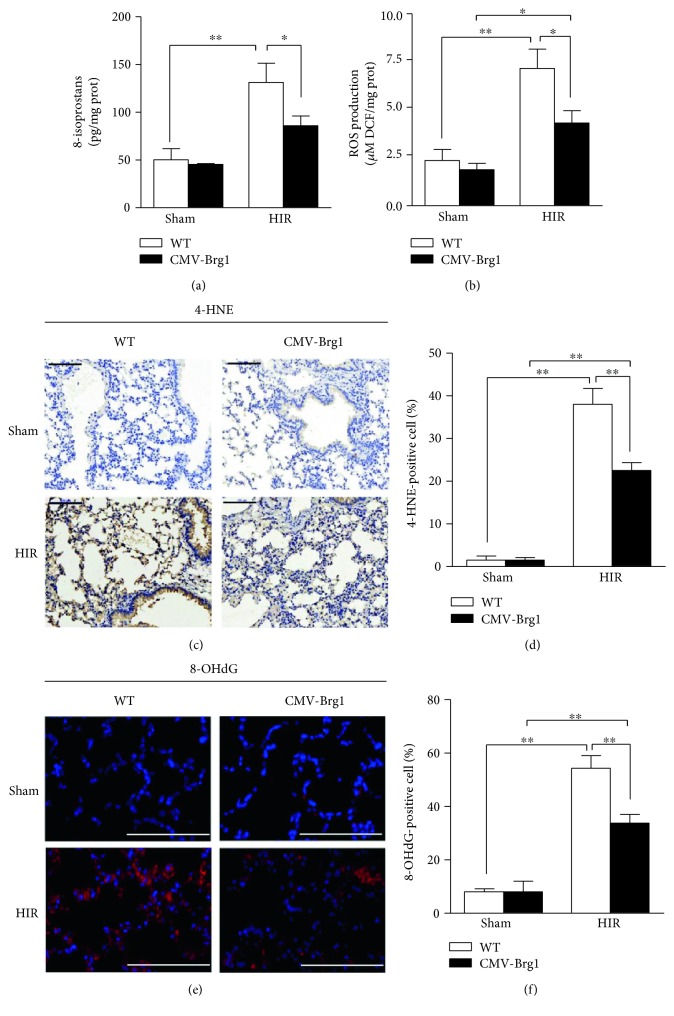
Overexpression of Brg1 protects against oxidative stress induced by HIR. (a, b) Lung 8-isoprostane and ROS levels. (c, d) Immunohistochemical staining (×200) of lung 4-hydroxynonenal (4-HNE) (brown) and densitometric analysis showed that the 4-HNE-positive cells were significantly reduced in the CMV-Brg1 mice than in the WT mice at 6 h after HIR. (e, f) Immunofluorescence staining (×200) of lung 8-hydroxy-2-deoxyguanosine (8-OHdG) and densitometric analysis showed that the relative fluorescence intensity was significantly decreased in the CMV-Brg1 mice compared to the WT mice at 6 h after HIR. The 8-OHdG-positive cells were stained red, and the nuclei of tissue sections were counterstained blue by 4,6-diamidino-2-phenylindole. ^∗^*P* < 0.05; ^∗∗^*P* < 0.01.

As shown in Figures 4(a) and 4(b), compared with the sham group, the levels of 8-isoprostane and ROS in the lung tissues of the WT mice were significantly increased after HIR; similarly, the relative fluorescence intensity of 8-OHdG (a marker of DNA oxidative damage) and the number of cells positive for 4-HNE (a marker of lipid peroxidation) were significantly increased in the WT HIR group (Figures 4(c), 4(d), 4(e), and 4(f)). However, compared with the WT mice undergoing HIR, the expression of 8-iso-prostaglandin, ROS, 4-HNE, and 8-OHdG in the CMV-Brg1 mice was significantly decreased after HIR, showing that Brg1 overexpression can effectively suppress the oxidative damage induced by HIR.

### 3.3. Association of Brg1 Overexpression with Increased Nuclear Transfer of Nrf2

Next, we explored the role of Brg1 on Nrf2 in ALI induced by HIR. As shown in Figures 2(a) and 2(c), after the process of HIR, Nrf2 protein expression in the WT mice was not significantly increased, compared with the sham group. In contrast, Nrf2 protein expression in the CMV-Brg1 mice after the process of HIR was significantly increased.

The CMV-Brg1 mice expressed significantly more Nrf2 protein after HIR than the WT mice (Figures 2(a) and 2(c)). As shown by the immunohistochemistry results presented in Figures 2(d) and 2(e), HIR significantly increased the immunohistochemical staining intensity of Nrf2-positive cells in lung tissue of the CMV-Brg1 mice at 6 h after HIR.

### 3.4. Association of Brg1 Overexpression with Activated Antioxidant and Decreased Inflammatory Factors

**Figure 5:**
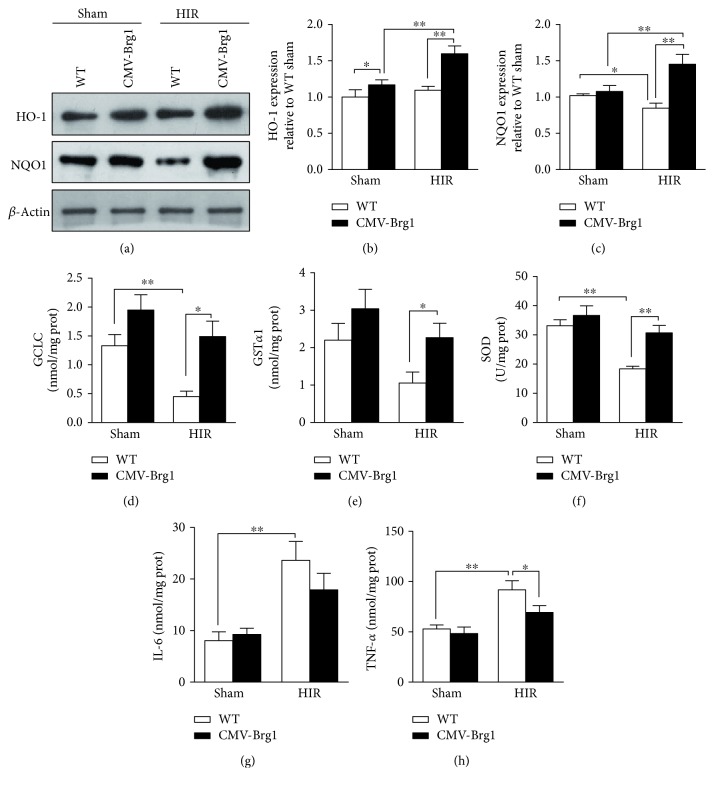
Association of Brg1 overexpression with activated antioxidant and decreased inflammatory factors. (a–c) the protein expression of HO-1 and NQO1 in the CMV-Brg1 and WT mice after HIR. (d–h) the concentrations of glutamate-cysteine ligase catalytic subunit (GCLC), glutathione S-transferase alpha 1 (GST*α*1), superoxide dismutase (SOD), interleukin-6 (IL-6), and tumor necrosis factor-*α* (TNF-*α*) in the CMV-Brg1 and WT mice after HIR. ^∗^*P* < 0.05; ^∗∗^*P* < 0.01.

As Nrf2 is known as a regulator of antioxidant enzymes, we compared the levels of enzymes between the WT and CMV-Brg1 mice. Compared with the sham group, the protein expressions of NQO1 and HO-1 in the WT mice after HIR were not significantly increased. In the CMV-Brg1 mice after HIR, both NQO1 and HO-1 protein expressions were significantly increased (Figures 5(a), 5(b), and 5(c)). Moreover, compared with the HIR WT mice, the HO-1 and NQO1 protein expressions in the CMV-Brg1 mice were significantly increased after HIR (Figures 5(a) and 5(b)). These data indicate that the protein expressions of HO-1 and Nrf2 after HIR might be regulated by Brg1, but with little relation to NQO1.

At the same time, we tested the changes in antioxidant enzyme levels, including GST*α*1, SOD, and GCLC. Compared with the sham groups, SOD, GCLC, and GST*α*1 in the WT mice after HIR were reduced significantly. However, the decreased magnitude of SOD, GCLC, and GST*α*1 in the CMV-Brg1 mice was less than that in the WT mice after HIR, indicating that Brg1 overexpression might play a crucial role in prompting the activation and expression of antioxidant enzymes (Figures 5(d), 5(e), and 5(f)). Moreover, we observed an inflammatory response. As shown in Figures 5(g) and 5(h), compared with the sham groups, the levels of IL-6 and TNF-*α* were significantly increased after HIR in the WT mice, but not in the CMV-Brg1 mice. After HIR, the TNF-*α* level was significantly decreased in the CMV-Brg1 mice (*P* < 0.05 versus the WT mice).

## 4. Discussion

In the current study, we demonstrated that HIR can induce remote ALI accompanied by a significant increase of oxidative damage and a significant decrease of Brg1 expression. Furthermore, we revealed that overexpression of Brg1 attenuated lung injury after HIR, promoted Nrf2 nuclear translocation, and attenuated oxidative stress by activating the antioxidase system including HO-1, NQO1, SOD, GCLC, and GST*α*1.

It is well known that excessive ROS production is one of the main mechanisms involved in ALI after HIR [12, 18] and that antioxidant enzyme activation alleviates ALI induced by HIR [10, 30]. Acting like a transcriptional factor and cell defense response regulator, Nrf2 translocates into the nucleus following exposure to oxidative stress and activates the gene expression of many antioxidase and cytoprotective proteins, including GCL, HO-1, GPx, and NQO1 [22]. We have reported previously that HO-1 induction protects the lungs against ALI induced by liver transplantation in rats [31]. Recently, several studies have documented that upregulation of Nrf2/HO-1 plays a critical protective role in ALI induced by lipopolysaccharides or intestinal ischemia/reperfusion [23, 32, 33]. However, no studies have yet reported that Nrf2 and its downstream antioxidant enzymes can confer a protective effect against lung injury induced by HIR. Similar to earlier reports, we demonstrated that HIR induces significant oxidative stress by producing large amounts of ROS, 8-isoprostane, 4-HNE, and 8-OHdG. However, Nrf2 expression did not significantly increase until the ROS levels and lung pathological damage reached a peak at 6 h after reperfusion. Interestingly, once significantly activated, Nrf2 quickly promoted the expression of antioxidant enzymes and protected the lung from oxidative injury. Considering both the immediate oxidative stress and the inhibited induction of Nrf2, we assumed that upregulation of Nrf2 expression could play a critical role in alleviating lung injury after HIR.

Brg1, a subunit of the chromatin remodeling complex, has been demonstrated not only to regulate gene transcription and expression but also to play an important role in stem cell proliferation and differentiation in different tissues and cells in humans and animals [24, 34]. In addition, it has been shown to contribute to the process of disease development, such as lung tumorigenesis and cytokine response [24].

Reduction and loss of Brg1 expression have been related to dedifferentiation in lung cancers [35, 36]. Brg1 also has been found to act in concert with Fanconi anemia proteins to protect the promoters of antioxidant defense genes from oxidative damage [37]. In our experiments, we found that Brg1 was reduced after reperfusion, reaching a minimum at 6 h, while oxidative damage peaked. Interestingly, using transgenic CMV-Brg1 mice, we found that Brg1 overexpression promoted Nrf2 nuclear translocation, activated the downstream antioxidant system, and then alleviated post-HIR lung oxidative damage. Originally, Brg-1 was described as the factor responsible for selective HO-1 enzyme induction triggered by Nrf2 [25]. However, our findings indicated a more universal effect for Brg1: Brg1-promoted Nrf2 nuclear translocation mediates HO-1 induction; but the precise underlying mechanism remains to be determined. Therefore, the current study provides new potential therapeutic insights for scavenging ROS to combat HIR-mediated ALI.

It is worth noting that Brg1 interacted with Nrf2 to mediate HO-1 and NQO1 induction in order to counteract oxidative stress and activated antioxidases including GST, GCLC, and SOD. This finding was inconsistent with earlier reports showing that BRG1-mediated chromatin remodeling is essential for RNA polymerase II recruitment to the HO-1 promoter but not to the NQO1 promoter when subjected to oxidative stress in streptozotocin-induced diabetic rats [25, 38]. However, whether lung injury induced by HIR is aggravated in Brg1 gene deficiency still needs to be explored, especially by using Brg1 gene-deficient mice. These results will help to elucidate the role of Brg1 in lung protection. Moreover, whether other pathways can mediate Brg1 involvement in lung injury after HIR needs to be further investigated.

## 5. Conclusion

In conclusion, the present results demonstrated that HIR could induce remote lung injury accompanied by a significant increase of oxidative damage and a significant decrease of Brg1 expression at the early reperfusion time. Brg1 overexpression significantly protected the lung from HIR injury through Nrf2 activation and its downstream antioxidase activity, including HO-1, NQO1, SOD, GCLC, and GST*α*1. Though the exact mechanisms of the interaction of Brg1 and Nrf2-induced downstream antioxidases under HIR settings remain to be studied, upregulation of Brg1 may be a potential therapy to alleviate remote lung injury after HIR.
